# Supplementary material for: Freezing and piercing of in vitro asymmetric plasma membrane by α-synuclein
Source: Commun Biol. 2020 Mar 31;3:148. doi: 10.1038/s42003-020-0883-7 (PMC7109109; doi:10.1038/s42003-020-0883-7)
Supplement: Supplementary file 1 — Supplementary Information [file 42003_2020_883_MOESM1_ESM.pdf]

Supplementary information

Freezing and piercing of *in vitro* asymmetric plasma membrane by α-synuclein

Supplementary Figures

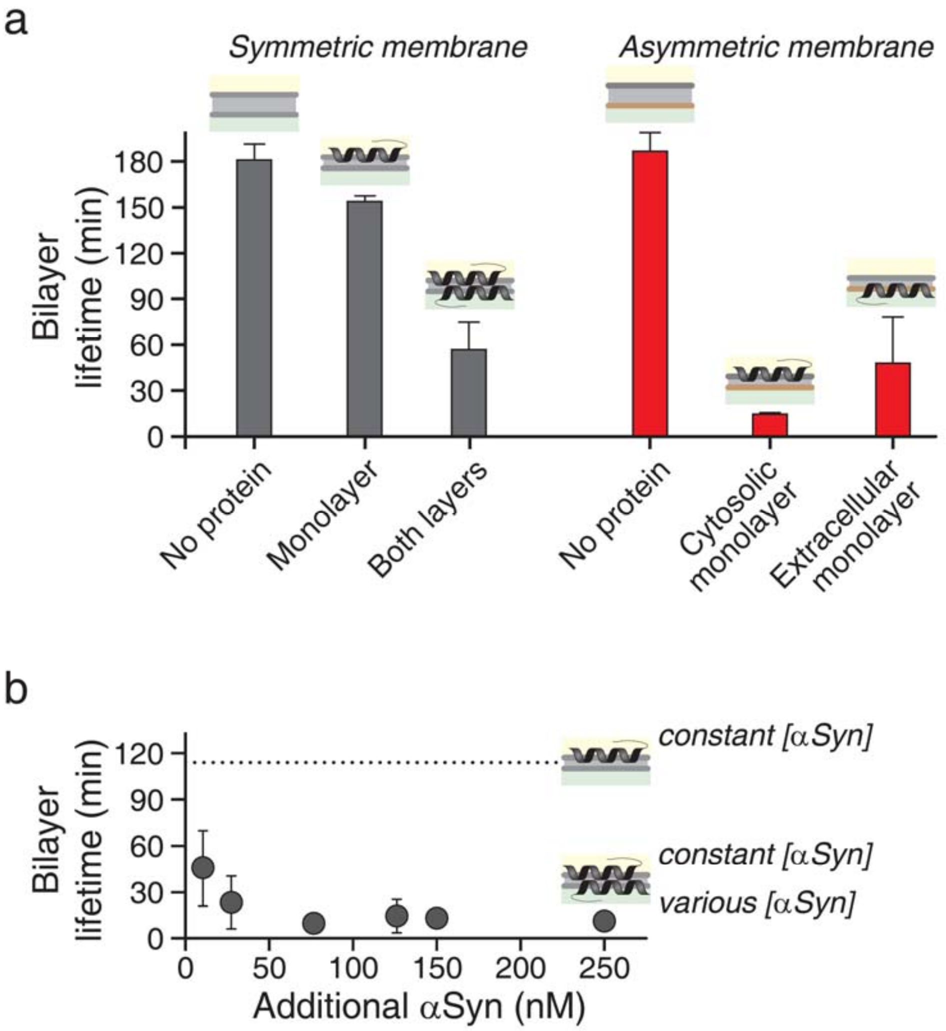

Supplementary Figure 1. (a) Comparison of the average membrane lifetime under various conditions: no protein and symmetric membrane, 25 nM αSyn in one channel and symmetric membrane, 25 nM αSyn in both channels and symmetric membrane, no protein and asymmetric membrane, 25 nM αSyn in the cytosolic channel and asymmetric membrane, 25 nM αSyn in the extracellular channel and asymmetric membrane. Red (resp. grey) leaflet indicates cytosolic (resp. extracellular) lipid composition. Error bars are standard errors of the mean. (b) Lifetime of symmetric membrane in the presence of αSyn in both leaflets. Cytosolic αSyn is kept at 60 nM in one channel and the desired αSyn concentration is injected in the other channel. Dashed line indicates the lifetime in absence of αSyn in the second channel. Error bars are standard errors of the mean.

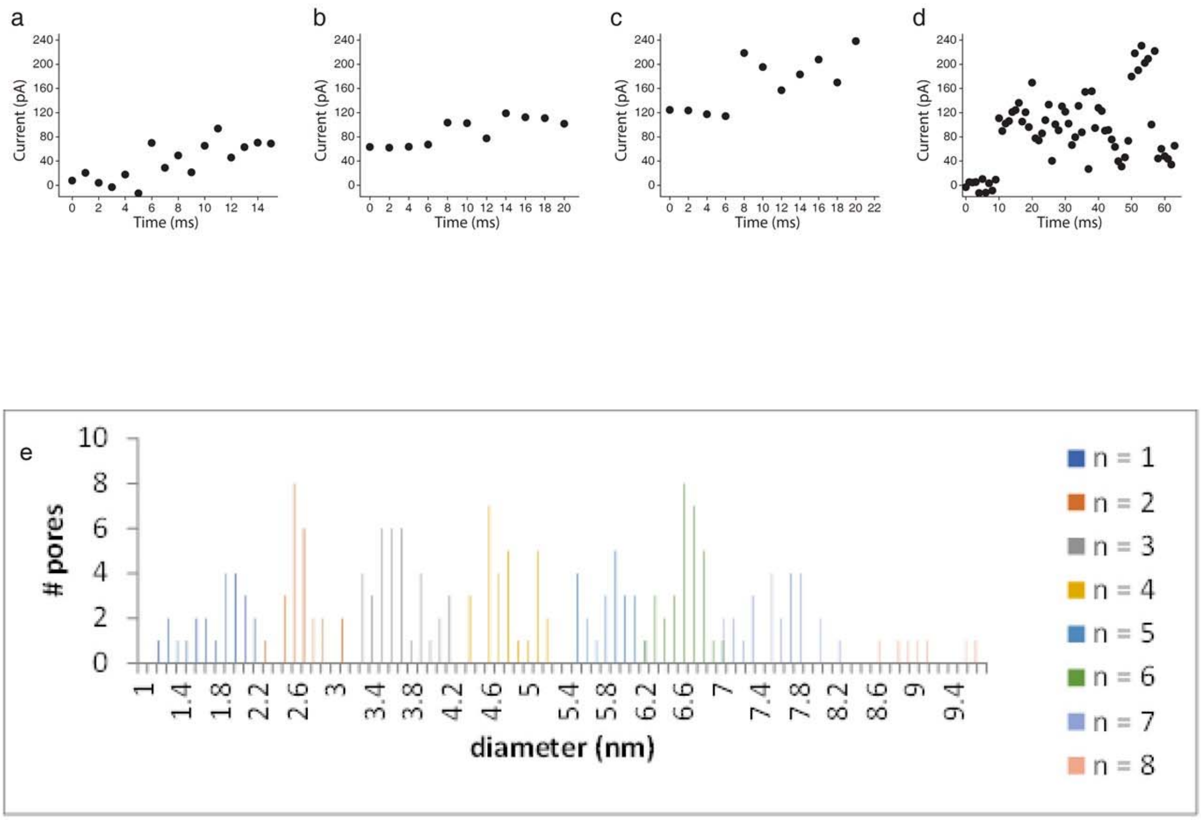

Supplementary Figure 2. Examples of current steps (pore opening and closing). Panels a to c represent jumps between steps 0 and 1, 1 and 2, and 2 and 3 respectively; panel d shows an example of expansion and reduction of a pore between states 0, 1, 2, and 3. These are from 4 different pores to show the reproducibility of the steps. The intensity values of the steps presented in Figure 3a-c are based on the distribution of these jumps and the plateau values. (e) Histogram of the number of observed pore vs. the diameter of the pore. For each individual pore, the diameter was obtained by averaging up to 10 points following the corresponding current jump. Each color indicates the number of steps that have been counted by following the whole current trace.

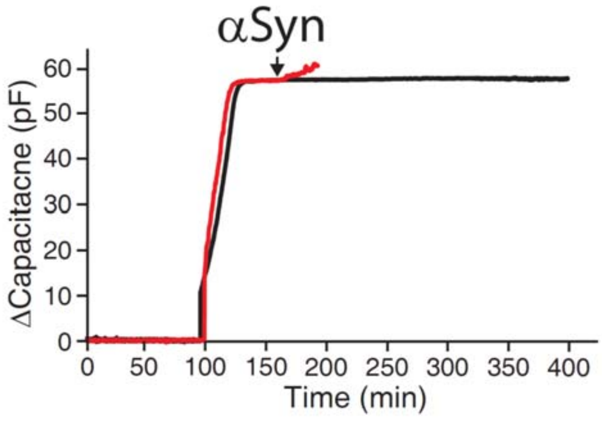

Supplementary Figure 3. Example of capacitance trace without switching to current mode. Injection of 100 nM αSyn is indicated by the arrow. The subsequent increase confirms that αSyn raises the apparent specific capacitance.

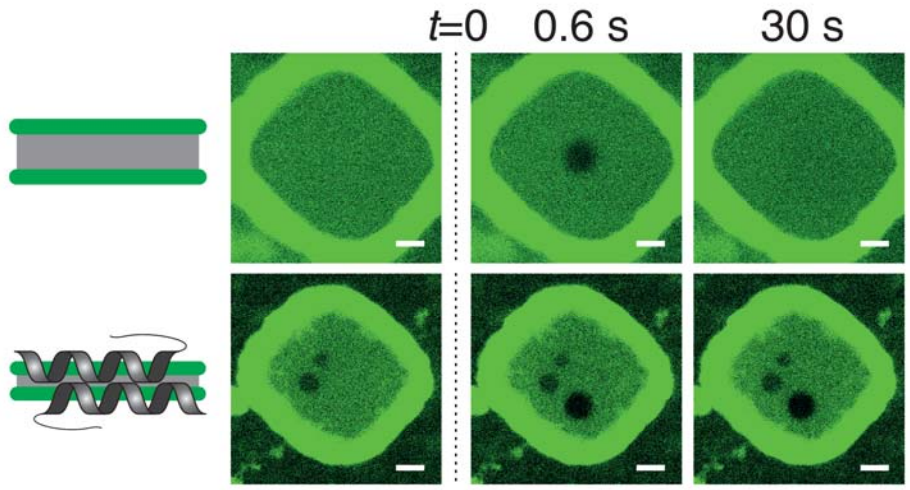

Supplementary Figure 4. Examples of fluorescence recovery after photobleaching. Membrane is labeled with fluorescent lipids. The successive panels show the membrane fluorescence before, 0.6 s and 30 s after bleaching a 20 μm disk. In the presence of αSyn, previously bleached areas corresponding to 10 and 15 μm disk diameters are still visible due to hindered fluidity. Scale bars, 20 μm.
