# Supplementary material for: Freezing and piercing of in vitro asymmetric plasma membrane by α-synuclein
Source: Commun Biol. 2020 Mar 31;3:148. doi: 10.1038/s42003-020-0883-7 (PMC7109109; doi:10.1038/s42003-020-0883-7)
Supplement: Supplementary file 6 — Description of Additional Supplementary Files [file 42003_2020_883_MOESM6_ESM.pdf]

Description of Additional Supplementary Files

File Name: **Supplementary Data 1**
Description: In sheet 1, Figure 1g, are provided the membrane lifetimes for various aSyn concentrations as presented in Figure 1g. Data in sheet 2, Figure 4a, represent the variation of capacitance with membrane area for protein-free membranes, membrane with aSyn and membrane with t-SNARE. Sheet 3, Figure 4d, provides the data for the FRAP experiments presented in Figure 4d.

File Name: **Supplementary Data 2**
Description: List of current jumps measured for steps 1 to 9. These data, except step 9, are plotted in Figures 3 b and c. Columns AL to BI are the data corresponding to supplementary Fig. 2e.
